# Distribution of Plantar Pressure in Soccer Players

**DOI:** 10.3390/ijerph18084173

**Published:** 2021-04-15

**Authors:** Arletta Hawrylak, Anna Brzeźna, Krystyna Chromik

**Affiliations:** 1 Department of Physiotherapy, University School of Physical Education, al. Ignacego Jana Paderewskiego 35, 51-612 Wrocław, Poland; krystyna.chromik@awf.wroc.pl; 2 Physiotherapist, Private Practice, 2 rue Jacques Rodallec, 56110 Gourin, France; brzezna@o2.pl

**Keywords:** men, soccer, baropodometry, biomechanics, training

## Abstract

(1) Background: The aim of this study was to evaluate differences in the static and dynamic distribution of foot pressure on the ground and to investigate the relationships between body mass index (BMI) and mean variables of plantar pressure between soccer players and their non-athlete peers. (2) Methods: The study involved 18 first-division Polish soccer players and 30 non-athlete physiotherapy students. The research experiment was conducted using the FreeMed platform. Basic descriptive statistics were calculated to summarize the variables. Additionally, in the static and dynamic tests, Spearman’s rank correlations between body mass index (BMI) and plantar load were calculated. (3) Results: Statistically significant differences between groups were observed in the loading of the dominant limb. A statistically significant correlation between BMI and loading of both limbs was found in the static test and between BMI and loading of the dominant limb in the dynamic test. (4) Conclusions: The baropodometric mat used in our study helped determine the plantar pressure distribution of soccer players and their non-athlete peers. Correlation analysis revealed that BMI was only associated with the mean plantar pressure of the dominant limb in the control group. Further research on a larger group of athletes is needed to determine how much sporting activity may affect the development to modifications within feet in soccer players.

## 1. Introduction

Soccer is a demanding, high-intensity sport that requires considerable endurance, agility, speed and strength. The core component of moving in soccer is running, with an average distance of 10 km covered during a match. Running in soccer is highly a cyclical and unstructured. It comprises quick accelerations, slow jogs, jumps, lunges and instantaneous changes in direction with and without the ball. Hence, involvement in soccer on a professional level may induce significant loads on the osteoarticular and muscle systems not seen in typical running modalities [1].

Due to the nature of the game of soccer, players often struggle with various injuries that most often occur during a match. The most common ones include muscle strains, tendon injuries, joint sprains and, less commonly, fractures. The anatomical regions where most injuries occur are the knee joint, ankle joint, thigh, groin, hip joint and foot [2,3]. Combined with multiple repetitions, significant overloads can cause additional injuries. When comparing soccer-specific moves with regular running, increased loading was observed in the forefoot region during sprinting and the lateral side of the foot during dribbling [4]. The characteristic distribution of the pressure and the unique loading in individual foot zones are observed in specific movements. The particular model and high foot pressure is observed during normal running. The lowering of the center of gravity in the midfoot occurs in sprinting during the first and second half-straight, while the lateral side of the foot is the most loaded during the kick [4]. An individual playing professional soccer is required to continually develop motor skills, technique and tactics in training and during competitions, which can lead to injuries [5]. The most prone to overload are tall soccer players, those who spend more time on the pitch during a match and those who are required to play in defensive positions, with frequent contact with the opponent, often running at full speed with changes in running direction and performing quick accelerations [5]. In addition, to the aspects of mechanical damage to the musculoskeletal system that exposes players to injury risk, environmental aspects are also often emphasized, i.e., the volume and quality of training, the way the playing field is prepared and the fouls committed by players during the game [6]. The aim of this study was to evaluate differences in the static and dynamic distribution of foot pressure on the ground and to investigate the relationships between body mass index (BMI) and mean variables of plantar pressure between soccer players and their non-athlete peers. The experiment consists of two tests: in static and dynamic.

## 2. Materials and Methods

### 2.1. Subjects

The study involved 18 first-division Polish soccer players from the Śląsk Wrocław club and 30 non-athlete physiotherapy students from the Faculty of Physiotherapy at the University School of Physical Education in Wrocław, Poland. Basic anthropometric characteristics for both groups are presented in Table 1.

The inclusion criteria were written informed consent to participate in the experiment and no professional training in any sport (control group). The exclusion criteria were failure to provide written informed consent to participate in the experiment, professional training in a sport (control group) and musculoskeletal injuries.

Written informed consent was obtained from all participants. The experiment was conducted in accordance with the Declaration of Helsinki [7], with the consent of the Senate’s Research Bioethics Commission at the University School of Physical Education in Wrocław, Poland.

### 2.2. Procedures

The experiment was conducted using the FreeMed platform for the measurement of ground reaction forces with FreeStep software. The device analyses which part of the foot presses harder on the ground while standing and walking. Both in the static and dynamic tests, the participant stood in the measurement area, with feet parallel to each other at one height and without footwear. After assuming the correct position, the measurement was started by switching on the start button. The test lasted 5 s and, by clicking the Close button, the collected data were automatically saved. Furthermore, in the dynamic test, the participant was instructed to move freely across the mat to its end, make a turn and return. Natural gait was recorded, with the alternating work of the upper limbs and eyes looking straight ahead. After the platform recorded three take-offs for the left and right foot, the test was terminated by pressing the Stop button. Autosaving of the collected data occurred when the Close button was pressed [8,9]: www.koordynacja.com.pl (2009) (accessed on 9 April 2021). Limb dominance was determined using the methodology developed by Tichy and Belacek [10]. The following clinical criteria extrapolated from the data separately for the dominant and non-dominant limb were evaluated in both tests: (1) Calcaneus angle corresponding to the gamma heel angle as an angular characteristic of foot geometry in the static condition. The limits of the range of motion of this angle are 15–18° [11]. (2) Load distribution in the forefoot and rearfoot in the dominant and non-dominant limbs. (3) Distribution of the load in the medial and lateral parts of the foot in the dominant and non-dominant limbs. (4) Mean foot loading in the dominant and non-dominant limbs.

The experiments were conducted one after another without rest. The subjects performed the tests without footwear. The test was performed in a closed room with access to daylight, in the research laboratory of the Department of Kinesitherapy of the University School of Physical Education in Wrocław. 

On the test day, the subjects did not do any increased physical activity. Measurements were conducted during the autumn round of soccer league games.

### 2.3. Statistical Analyses

Statistical analysis was performed using Statistica v. 9 PL software (Stat soft, Poland). Basic descriptive statistics were calculated to summarize the variables. The normality of the data set was confirmed using the Shapiro–Wilk normality test. Student’s t test was used to make between-group comparisons. Additionally, in the static and dynamic test, Spearman’s rank correlations between body mass index (BMI) and plantar load were calculated, with statistical significance assumed at *p* ≤ 0.05.

## 3. Results

### 3.1. Static Foot Assessment

The differences between the groups were not statistically significant, the calcaneus angle of the dominant and non-dominant limb was greater in the control group (Table 2). Based on the results from Table 3, no statistically significant differences were found between the groups, higher forefoot load values in both limbs were observed in the group of soccer players (Table 3). No relationship was observed between BMI and loading of the dominant and non-dominant limbs in the static test in both groups (Table 4).

### 3.2. Dynamic Foot Assessment

It was found in the dynamic test that higher forefoot loading and lower rearfoot loadings occurred in soccer players in both lower limbs. There were no statistically significant differences in the parameters studied between the groups (Table 5). The differences in lateral and medial loads on the dominant limb were statistically significant between the groups. In the group of soccer players, the side of the foot was more loaded in both the dominant and non-dominant limbs, while in the control group, a greater load was observed in the medial part (Table 6). A statistically significant correlation was also observed between BMI and loading of the dominant limb in the control group (Table 7).

## 4. Discussion

Soccer players are required to constantly develop their motor skills, technique and tactics, which can lead to a significant overload of the musculoskeletal system [5]. Most researchers focus mainly on the body posture analysis of athletes. Functional fitness and the associated shape of foot arches have not been sufficiently studied. It is foot defects that can alter gait biomechanics, resulting in the ineffective performance of motor activities and a significant load on the athletes’ locomotor system [2]. As one of the contact sports, soccer can lead to injuries, especially in the foot and ankle, amounting to 20% of all injuries [12,13]. According to Bastos et al. and Baron et al., soccer players who play in defensive positions, with frequent contact with the opponent, those who run at full speed with changes in directions and those who perform rapid accelerations at their position on the pitch are particularly vulnerable to musculoskeletal injuries [5,13]. Improved performance, optimal preparation of players for performing sprints and changes of direction and reduced risk of injury can be achieved with properly designed warm-up routines before training and games [13,14]. Soccer is a sport that requires players to position their feet properly. Based on the results of their research, the authors observed a significantly greater load on the lateral side of the foot and the forefoot in a group of players in both the dominant and non-dominant limbs and both in dynamic and static tests. These results are also consistent with those presented by Grabara, who examined soccer players and reported genu varum, foot positioning on lateral edges and a significant forefoot loading in the subjects [12]. In contrast, Carl et al. demonstrated a relationship between dedicated soccer footwear and loading in specific parts of the foot. They obtained statistically significant results in the parameter for lateral foot loading in the dominant and non-dominant limbs [15]. The study by Eils et al. drew attention to the characteristic distribution of the foot pressure zones depending on the specific movement performed by the footballer. The middle of the foot is loaded more when receiving the ball. During kicking, more load is on the lateral part, while during sprint on the forefoot [4]. This is also supported by the study of Wong et al. who showed that during jumping and kicking, foot loading is higher compared to normal running and the highest pressure was found in the medial area of the sole of the foot [3]. The greater load on the side of the foot in the group of soccer players in our study may be the cause of the frequent occurrence of ankle sprain, which is one of the most common injuries in soccer. These findings are supported by the study of Woods et al., who also found a high incidence of injury in the ankles and the associated strain of the lateral ligaments in the lower leg, which may be attributable to habitual loading of the side of the foot [16]. The publication also analyzed the correlation between BMI and mean foot loading in both study groups in static and dynamic tests. A correlation was only demonstrated in the control group in the dominant limb, which may be linked to the center of gravity shifted forward in the study group and the poorer central postural stability in these individuals. According to Woods et al., due to better-developed core muscles and strengthening of the foot arches during training, soccer players are characterized by a more even distribution of the load in the lower limbs [16]. In their study, Puszczałowska-Lizis et al. also failed to demonstrate a relationship between BMI and forefoot loading. They concluded that the fact that most of the participants had normal body weight and a correctly shaped foot may have influenced the results [17]. In his research, Hills emphasized the compensation that occurs due to the excessive loading with body weight, resulting in increased forefoot width and lowering of the longitudinal arch of the foot [18]. Similar research was conducted by Tsung et al., who found that doubling the body weight can increase the support surface [19]. A study by Souza da Rocha and Kaplan et al. and Clark and Chaudhry confirmed that excessive body weight can lead to reduced sensation in the feet and their greater pressure on the ground, which may contribute to a greater risk of injury [20,21,22,23]. A study by Campy et al. reported that high body mass index (BMI) and high body fat may have an adverse effect on normal movement patterns [24]. The issues addressed in the paper may allow for the implementation of preventive measures and early diagnosis of musculoskeletal disorders [25].

Competition in football during matches and training requires players to develop high technical and tactical skills and the ability to play under high stress [26,27]. Studies by Wong and Hong and Murphy et al. showed that more injuries occur during a match than during training [28,29].

On the other hand, football as a team sport teaches cooperation and can provide psychological comfort to players.

## 5. Conclusions

The baropodometric mat used in our study helped determine the plantar pressure distribution of soccer players and their non-athlete peers. Correlation analysis revealed that BMI was only associated with the mean plantar pressure of the dominant limb in the control group. Further research on a larger group of athletes is needed to determine how much sporting activity may affect the development to modifications within feet in soccer players.

### Practical Applications

The innovative approach to the problem of incorrect foot loading in soccer players presented in this paper may contribute to the reduction of injury rates in this group of athletes, because the overload due to incorrect foot positioning can, through the biokinematic chain, be transferred to the entire locomotor system. The results of this research may be used by coaches and athletes to ensure the safe development of the musculoskeletal system of the athletes and allow for complementing current training programs.

## Figures and Tables

**Table 1:** Anthropometric characteristics of the groups.

Variable	Soccer Group	Control Group
Age (years)	19.7 ± 1.52	21.3 ± 1.65
Body height (m)	178.9 ±6.65	178.8 ± 4.68
Body mass (kg)	72.3 ± 6.28	74.36 ± 8.47
BMI (kg/m^2^)	22.58 ± 1.13	23.22 ± 2.14

**Table 2:** Mean ($\bar{x}$), standard deviation (SD) and t-test results for the calcaneus angle measures in the dominant and non-dominant limbs in the static test.

Variable	Normative Range	Soccer Group		Control Group		Soccer–Control	
		$\bar{x}$	*SD*	$\bar{x}$	*SD*	*t*	*p*
Calcaneus angle dominant limb (°)	15–18	10.94	5.63	12.70	5.85	−1.01	0.31
Calcaneus angle non-dominant (°)	15–18	10.88	5.66	11.13	6.33	−0.13	0.89

**Table 3:** Mean ($\bar{x}$), standard deviation (SD) and t-test results for plantar pressure distribution in the dominant and non-dominant limbs (forefoot/rearfoot loading) in the static test.

Variable	Soccer Group		Control Group		Soccer–Control	
	$\bar{x}$	*SD*	$\bar{x}$	*SD*	*t*	*p*
Forefoot loading, dominant limb (%)	53.61	12.27	52.40	9.77	0.37	0.70
Rearfoot loading, dominant limb (%)	46.38	12.27	47.60	9.77	−0.37	0.70
Forefoot loading, non-dominant limb (%)	51.83	15.86	49.36	8.19	0.71	0.48
Rearfoot loading, non-dominant limb (%)	48.16	15.86	50.63	8.19	−0.71	0.48

**Table 4:** Spearman’s rank correlation coefficients of BMI (body mass index) and load in the dominant and non-dominant limbs in the static test in both study groups.

Variable Pairs	Spearman’s Rank Correlation			
	n- Valid	Spearman’s Rho	t(N-2)	*p*
BMI and dominant loading (soccer group)	18	0.18	0.76	0.45
BMI and dominant loading (control group)	30	0.04	0.24	0.80
BMI and non-dominant (soccer group)	18	0.17	0.70	0.49
BMI and non-dominant (control group)	30	0.03	0.18	0.85

**Table 5:** Mean ($\bar{x}$), standard deviation (SD) and t-test results for plantar pressure distribution in the dominant and non-dominant limbs (forefoot/rearfoot loading) in the dynamic test.

Variable	Soccer Group		Control Group		Soccer–Control	
	$\bar{x}$	*SD*	$\bar{x}$	*SD*	*t*	*p*
Forefoot loading, dominant limb (%)	59.00	7.49	58.43	6.55	0.27	0.78
Rearfoot loading, dominant limb (%)	41.00	7.49	41.90	6.46	−0.43	0.66
Forefoot loading, non-dominant limb (%)	58.27	6.27	58.06	6.86	0.10	0.91
Rearfoot loading, non-dominant limb (%)	41.72	6.27	41.93	6.86	−0.10	0.91

**Table 6:** Mean ($\bar{x}$), standard deviation (SD) and t-test results for plantar pressure distribution in the dominant and non-dominant limbs (medial/lateral loading) in the dynamic test.

Variable	Soccer Group		Control Group		Soccer–Control	
	$\bar{x}$	*SD*	$\bar{x}$	*SD*	*t*	*p*
Medial foot loading, dominant limb (%)	45.66	4.02	50.23	5.43	−3.05	0.003 *
Lateral foot loading, dominant limb (%)	54.33	4.02	49.76	5.43	3.05	0.003 *
Medial foot loading, non-dominant limb (%)	47.77	5.07	58.06	6.86	−0.54	0.58
Lateral foot loading, non-dominant limb (%)	52.22	5.07	41.93	6.86	0.54	0.58

* p ≤ 0.05.

**Table 7:** Spearman’s rank correlation coefficients of BMI (body mass index) and load in the dominant and non-dominant limbs in the dynamic test in both study groups.

Variable Pairs	Spearman’s Rank Correlation			
	n- Valid	Spearman’s Rho	t(N-2)	*p*
BMI and dominant loading (soccer group)	18	0.22	0.91	0.37
BMI and dominant loading (control group)	30	0.40	2.32	0.02 *
BMI and non-dominant (soccer group)	18	−0.22	−0.91	0.37
BMI and non-dominant (control group)	30	0.24	1.33	0.19

* *p* ≤ 0.05.

## Data Availability

Data confirming the reported results can be found at the Faculty of Physiotherapy of the Academy of Physical Education in Wrocław, al. Ignacego Jana Paderewskiego 35, building no. P4.
